# Iodine containing porous organosilica nanoparticles trigger tumor spheroids destruction upon monochromatic X-ray irradiation: DNA breaks and K-edge energy X-ray

**DOI:** 10.1038/s41598-021-93429-9

**Published:** 2021-07-14

**Authors:** Yuya Higashi, Kotaro Matsumoto, Hiroyuki Saitoh, Ayumi Shiro, Yue Ma, Mathilde Laird, Shanmugavel Chinnathambi, Albane Birault, Tan Le Hoang Doan, Ryo Yasuda, Toshiki Tajima, Tetsuya Kawachi, Fuyuhiko Tamanoi

**Affiliations:** 1 Institute for Integrated Cell-Material Sciences, Institute for Advanced Study, Kyoto University, Kyoto, Japan; 2 Kansai Photon Science Institute, Quantum Beam Science Research Directorate, National Institutes for Quantum and Radiological Science and Technology, Hyogo, Japan; 3 Center for Innovative Materials and Architectures, Vietnam National University-Ho Chi Minh City, Ho Chi Minh City, Vietnam; 4 Department of Physics and Astronomy, University of California, Irvine, CA USA

**Keywords:** Cancer, Nanoscience and technology

## Abstract

X-ray irradiation of high Z elements causes photoelectric effects that include the release of Auger electrons that can induce localized DNA breaks. We have previously established a tumor spheroid-based assay that used gadolinium containing mesoporous silica nanoparticles and synchrotron-generated monochromatic X-rays. In this work, we focused on iodine and synthesized iodine-containing porous organosilica (IPO) nanoparticles. IPO were loaded onto tumor spheroids and the spheroids were irradiated with 33.2 keV monochromatic X-ray. After incubation in CO_2_ incubator, destruction of tumor spheroids was observed which was accompanied by apoptosis induction, as determined by the TUNEL assay. By employing the γH2AX assay, we detected double strand DNA cleavages immediately after the irradiation. These results suggest that IPO first generate double strand DNA breaks upon X-ray irradiation followed by apoptosis induction of cancer cells. Use of three different monochromatic X-rays having energy levels of 33.0, 33.2 and 33.4 keV as well as X-rays with 0.1 keV energy intervals showed that the optimum effect of all three events (spheroid destruction, apoptosis induction and generation of double strand DNA breaks) occurred with a 33.2 keV monochromatic X-ray. These results uncover the preferential effect of K-edge energy X-ray for tumor spheroid destruction mediated by iodine containing nanoparticles.

## Introduction

Irradiation of high Z elements such as gadolinium, iodine, gold and silver with X-rays causes photoelectric effects that include the release of Auger electrons^1, 2^. This requires the use of X-rays that have energy levels at or higher than the K-edge energy of the element irradiated. The process involving electron release starts with the absorption of the X-ray energy by K-shell electrons resulting in their release causing destabilization of the atom. This is corrected by the movement of outer shell electrons to the K-shell releasing energy that is received by other electrons. Thus, a cascade of electron release occurs. Since the Auger electrons have strong DNA damaging and cell killing effects with the short mean path from the atom that absorbed the X-ray photon, this approach has been explored as a potential cancer therapy that can be termed the Auger therapy^2–4^. Initial studies used nucleotide precursors to place high Z element on DNA. Subsequently, photon-activation therapy (PAT) has been developed leading to a variety of studies using cancer cells and animal models^5–13^.

Nanoparticles containing high Z elements such as gold, gadolinium and iodine have been developed^14–22^. These nanosized particles (less than 300 nm diameter) have a number of properties that are beneficial for the Auger therapy. They are easily taken up into cancer cells and are localized at the periphery of cell nucleus, increasing the chance to damage cancer cell DNA^23^. In addition, the nanoparticles have the potential to accumulate in the tumor due to passive as well as active mechanisms. Indeed, in vivo, the high vascularization and porosity of the blood vessel wall lead to preferential accumulation of nanoparticles in the tumor cells compared to healthy cells. This phenomenon, called Enhanced Permeability and Retention effect (EPR effect) has been widely used to target preferentially cancer cells and surface modification can also be performed as an active targeting strategy^24–26^. High Z element loaded nanoparticles were shown to enhance radiation sensitivity in cultured cells and in animal models when irradiated with monochromatic X-ray^12–16^. Enhancement of radiation therapy was observed when conventional X-ray sources were used and some nanomaterials such as gadolinium loaded nanoparticles are evaluated in clinical studies^16^.

Recently, we developed gadolinium loaded mesoporous silica nanoparticles (Gd-MSN) and demonstrated their ability to destruct tumor spheroids upon irradiation with monochromatic X-rays^18^. Tumor spheroids are three-dimensional tumor mass and this makes the diffusion of nanomaterials and X-ray penetration more representative of the in vivo conditions than cancer cell assays. The tumor spheroid model can be modified by the inclusion of other types of cells such as fibroblasts and macrophages to produce tumor organoids that mimic tumor microenvironment^27^. We have shown that Gd-MSN are efficiently taken up into the spheroids and distributed throughout the spheroids^18^. Exposure of these tumor spheroids with synchrotron generated monochromatic X-rays with a narrow band width having an energy of 50.25 keV resulted in almost complete destruction of spheroids and this was dependent on the presence of gadolinium. Strikingly, the destruction did not occur with a monochromatic X-ray having an energy of 50.0 keV, demonstrating a sharp dependence on the K-edge energy of gadolinium. Furthermore, effect of 50.4 keV X-ray was less than that observed with 50.25 keV X-ray, raising the possibility that the optimum effect can be observed with the K-edge energy X-ray.

In this work, we utilized the above tumor spheroids assay and evaluated the effect of a new nanomaterial, iodine-containing porous organosilica nanoparticles (IPO). Iodine is a more economical high Z element than gold or gadolinium. In addition, iodine has a K-edge energy of 33.2 keV which is much lower than that of gadolinium or gold. Monochromatic X-rays having energy lower than 50 keV may be generated by the use of a compact X-ray source^28^. Iodine is used as an enhancing agent for CT scan^29, 30^. Finally, it is possible to incorporate iodine into porous silica nanoparticles by using a precursor that contains iodine. We devised a synthesis method that combines the iodine-containing precursor with organosilica precursors that were previously used for organosilica nanoparticles^31–33^. These nanoparticles are relatively stable and possess surface that can be modified^33–35^. We report synthesis and characterization of iodine containing porous organosilica nanoparticles (IPO). These nanoparticles were loaded efficiently onto tumor spheroids. Irradiation of IPO-loaded tumor spheroids with a monochromatic X-ray of 33.2 keV energy resulted in tumor spheroid destruction. The spheroid destruction was due to apoptosis induction. We also present evidence that double strand DNA breaks are induced immediately after the irradiation with a monochromatic X-ray of 33.2 keV. These effects were not observed when a monochromatic X-ray of 33.0 keV energy was used and were less pronounced when 33.4 keV X-ray was used. Finally, monochromatic X-rays with energies differing 0.1 keV were used. Our results demonstrate that an optimum effect on tumor spheroid destruction can be observed with a K-edge energy X-ray.

## Results

### Synthesis and characterization of iodine containing porous organosilica nanoparticles (IPO)

Our overall plan of this work is to first synthesize iodine containing nanoparticles called IPO, load them onto tumor spheroids and then irradiate the spheroids with synchrotron generated monochromatic X-rays and investigate destruction of tumor spheroids. The work relies on two of our abilities, one is to synthesize iodine containing nanoparticles and the other is our ability to generate monochromatic X-rays with narrow band width enabling examination of the effect of X-rays with differing energy levels.

**Figure 1:**
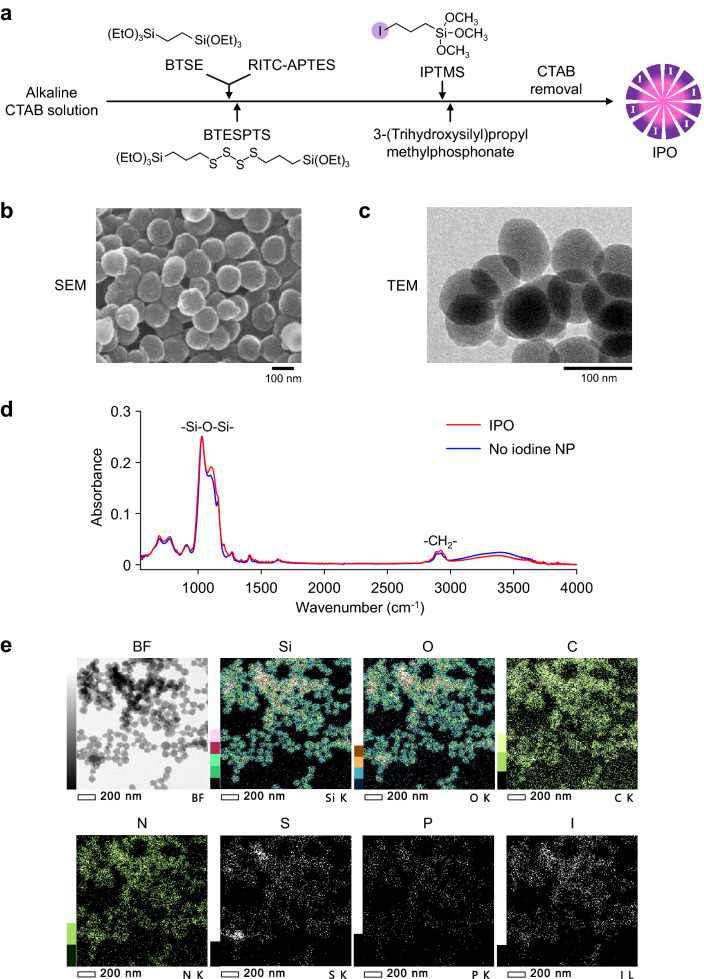
Synthesis and electron microscopy images of IPO nanoparticles. (**a**) Synthetic scheme of IPO. BTESPTS, bis[3-(triethoxysilyl)propyl]tetrasulfide; BTSE, 1,2-bis(triethoxysilyl)ethane; IPTMS, (3-iodopropyl)trimethoxysilane. SEM (**b**) and TEM (**c**) of IPO. (**d**) FT-IR analysis of IPO. (**e**) STEM-EDX with elemental mapping of Si, O, C, N, S, P and I of IPO. *BF* bright field.

The IPO synthesis is based on our previous synthesis of nanoparticles that employs the use of organoalkoxysilane precursors, bis[3-(triethoxysilyl)propyl] tetrasulfide (BTESPTS) and 1,2-bis(triethoxysilyl)ethane (BTSE)^32, 33^. Iodine precursor ([3-iodopropyl]trimethoxysilane—IPTMS) was co-condensed together with BTESPTS and BTSE to make porous organosilica nanoparticles that contain iodine atoms and tetrasulfide bonds within the network of the nanoparticles. Cetyltrimethylammoninum bromide (CTAB) was used as a surfactant to promote formation of pores in the nanoparticle. Phosphonate moieties were added to enhance dispersion in solutions, and this was carried out by using 3-(trihydroxysilyl) propyl methyl phosphonate. Rhodamine-B was also added during the synthesis to make the nanoparticles fluorescent. This dye was chosen for its capacity to emit in the range of observation of confocal microscopy and for complementarity with the system used in this study: Hoechst dye for nucleus observation (blue), green fluorescent protein for tumors detection and rhodamine-B for nanoparticle. Finally, the rhodamine-B isocyanate can be easily incorporated in the silica network via the use of APTES. An overall scheme for the synthesis of IPO is shown in Fig. 1a. Control nanoparticles without iodine, called “no iodine NP”, were synthesized without the addition of iodine precursor.

Scanning electron micrographs (SEM) of the nanoparticles synthesized are shown in Fig. 1b. Uniform size nanoparticles with an average diameter of 80 nm are consistent with TEM observations (Fig. 1c). DLS measurement showed average size of 80 nm (Supplementary Fig. S4). The nitrogen adsorption–desorption isotherm (Supplementary Fig. S3) reveals the presence of a small quantity of micro and mesopores, also reflected by a specific surface area of 67 m^2^ g^−1^. In addition, the zeta potential study showing the modification of the isoelectric point of the NP compared to bare NP (Supplementary Fig. S2) is consistent with a successful phosphonate modification of both IPO and no iodine NP. Figure 1d shows the FT-IR spectra of IPO as well as no iodine NP. The Si–O–Si stretching band at 1032 cm^−1^, as well as the CH_2_ symmetrical and asymmetrical stretching bands at respectively 2890 and 2925 cm^−1^ coming from aliphatic silylated precursors such as BTSE, BTESPTS or IPTMS were identified. A small variation of the Si–O–Si band shape is observed for IPO compared with no iodine NP, but this may be explained by the use of the IPTMS silylated precursor for IPO. A small band observed at 1207 cm^−1^ with IPO can be tentatively attributed to the CH_2_-I wagging vibration^36^ but is more likely due to organosilica related band deformation by the use of IPTMS. Other iodine related vibration bands are hardly observable here as they would overlap with the very broad Si–O–Si band or are located out of our observation range^36^. The overall spectra (related to the common silsesquioxane network) of IPO and no iodine NP are similar. The FT-IR together with the zeta potential measurements suggests that they have a common chemical composition. SEM and TEM analysis also evidence that both NP have similar morphology (Supplementary Fig. S1). In “Supplementary Information S1”, we added a table to summarize the comparison of IPO and no iodine NP (Supplementary Table S1).

The scanning transmission electron microscopy-energy dispersive X-ray (STEM-EDX) analysis of IPO is shown in Fig. 1e. The presence of the characteristic atoms such as silicon (Si), oxygen (O) and carbon (C) that form the organosilica network of the nanoparticles as well as nitrogen (N) and sulfur (S) from the tetrasulfide bond, APTES and rhodamine-B were observed. In addition, phosphorus (P) and iodine (I) signals were detected. The presence of iodine in IPO was further confirmed by carrying out ICP-AES (inductively coupled plasma atomic emission spectroscopy) analysis which showed that the amount of iodine is 0.033 mg per mg of IPO (0.26 µmol of I per mg of IPO).

### IPOs are efficiently taken up into tumor spheroids and are uniformly distributed

**Figure 2:**
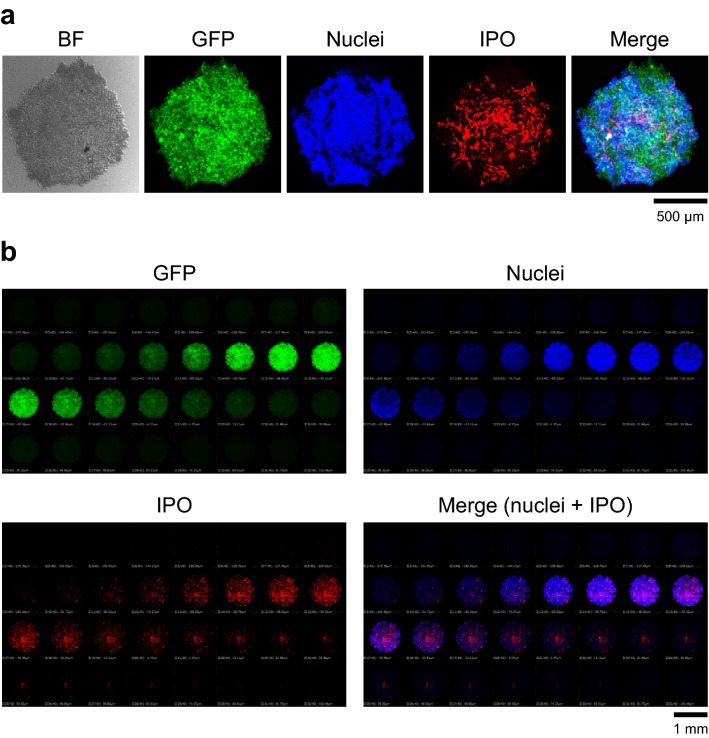
Uptake of IPO into tumor spheroids. (**a**) IPO nanoparticles were incubated with OVCAR8 tumor spheroids for 24 h, whose cells are engineered to express GFP. The tumor spheroids were washed and examined by confocal microscopy. The fluorescence of IPO labeled with rhodamine-B is shown in red. Nuclei were stained with Hoechst dye (blue). BF, bright field. (**b**) Fluorescence image at each focal plane.

IPOs were efficiently taken up into tumor spheroids and were distributed uniformly throughout the spheroids. Tumor spheroids were prepared by growing ovarian cancer OVCAR8 cells expressing GFP on a special plate that has hydrophilic surface and thus collects cells at the bottom of a well where spheroids are formed. The three-dimensional spheroids formed had a uniform size of approximately 0.5 mm × 0.5 mm and each spheroid contained 7.75 × 10^4^ cancer cells. They were incubated with IPO (rhodamine-B labeled) overnight (16–24 h). As can be seen in Fig. 2a, the spheroids exhibit green fluorescence due to GFP. The red fluorescence due to IPO can be seen throughout the spheroid, suggesting efficient distribution of IPO in the spheroid. In addition, we labeled cell nuclei with Hoechst that gave blue fluorescence. To provide more detailed information of the distribution of IPO in the spheroid, we carried out confocal microscopy to examine fluorescence at each focal plane (Fig. 2b). As can be seen, overlap of the red fluorescence of IPO was observed with green fluorescence of cancer cells and with blue fluorescence of cell nuclei at each focal plane, suggesting that the nanoparticles are generally localized in the cancer cells within the tumor spheroids.

### Tumor spheroid destruction by the exposure to monochromatic X-rays

**Figure 3:**
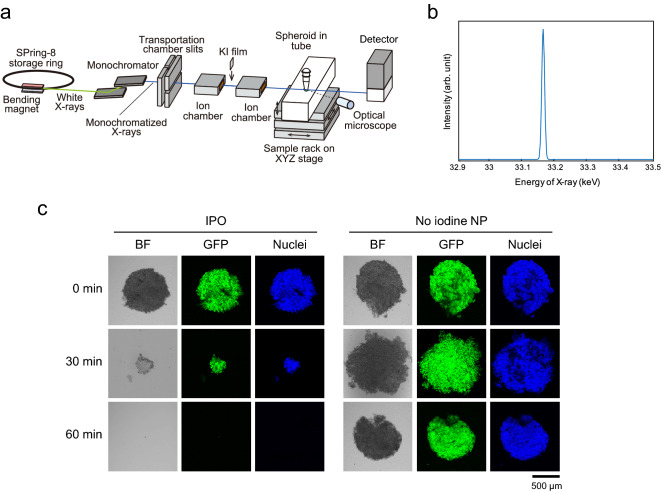
Monochromatic X-rays irradiation to tumor spheroids incubated with IPO. (**a**) Experimental setup for monochromatic X-ray irradiation. (**b**) Energy band width of monochromatic X-ray used in this study. (**c**) Effect of X-ray irradiation time on the destruction of tumor spheroids. OVCAR8 tumor spheroids incubated with IPO were irradiated with 33.2 keV monochromatic X-ray for the indicated time periods and destruction of tumor spheroids was examined. Tumor spheroids incubated with nanoparticles that do not contain iodine were used as no iodine controls (no iodine NP). GFP and nuclear staining were used to follow the destruction.

Irradiation of the IPO loaded tumor spheroids with monochromatic X-ray caused dramatic destruction of the spheroids. This experiment was carried out at a beamline BL14B1, SPring-8 synchrotron facility in Harima, Japan and the experimental setup is shown in Fig. 3a. Synchrotron radiation (SR) white X-rays from the bending magnet were led to a silicon double crystal monochromator to generate monoenergetic X-ray beam and the X-ray beam was shaped by using transport channel (TC) slits. The beam size was 1.4 mm × 0.7 mm at the sample position which is enough to cover the tumor spheroid that has the dimension of 0.5 mm × 0.5 mm. The X-ray beam intensities were monitored by ion chambers during the experiment. The SPring-8 storage ring was operated in top-up-mode in which X-ray intensity fluctuations in time were negligible. The transmitted X-rays were monitored with CCD camera to adjust the sample position. A sample rack is designed so that the spheroid is placed at the bottom of a tube and the irradiation position moves automatically to the next sample once the irradiation is completed. The photon flux at the sample position was calculated to be 3.9 × 10^8^ (photons s^−1^) using SPECTRA code^37^. The width of X-ray energy for one of the X-rays used is sharp as described in Fig. 3b. After the irradiation, the tumor spheroids were incubated in a CO_2_ incubator and kept at 37 °C up to 3 days and the spheroids were examined by visible and confocal microscopy. The reason for the inclusion of this incubation step is because cellular effect of X-ray irradiation takes time to manifest itself. The energy of monochromatic X-ray was set first at 33.2 keV which corresponds to the energy just above the K-absorption edge energy of iodine.

Figure 3c shows tumor spheroids after exposure to 33.2 keV monochromatic X-ray followed by incubation for 3 days. Tumor spheroids were seen with green fluorescence of GFP expressing cancer cells as well as by blue fluorescence of cell nuclei after staining with Hoechst dye. As can be seen, after 30 min irradiation, tumor spheroids loaded with IPO became much smaller and then were completely destructed after 60 min irradiation. In contrast, tumor spheroids without iodine (incubated with no iodine NP) appear intact even after 60 min irradiation. Thus, we could achieve dramatic tumor destruction and this occurs only in the presence of iodine. The irradiation with monochromatic X-ray does not have significant effect on tumor spheroids without iodine.

**Figure 4:**
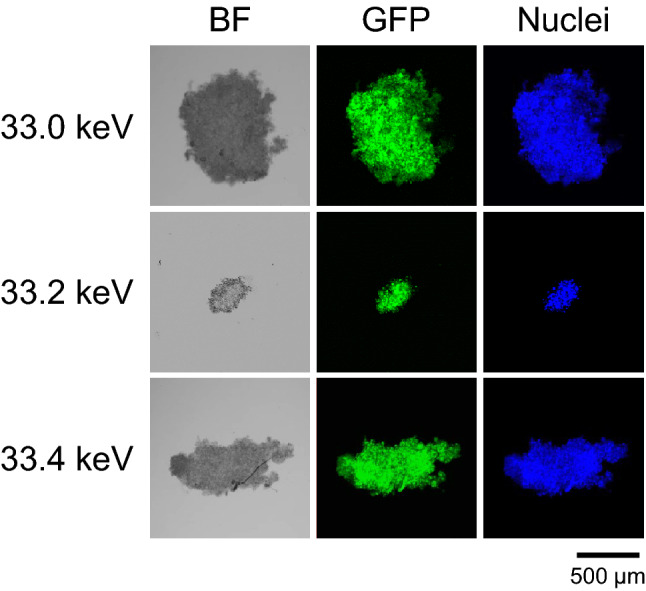
Energy dependence of tumor spheroid destruction. Tumor spheroids incubated with IPO were irradiated with three different monochromatic X-rays (33.0, 33.2 or 33.4 keV) for 30 min. GFP and nuclear staining were used to follow the destruction.

We have the ability to generate monochromatic X-ray beams with precise energy levels. The width of the X-ray beam is within 0.1% of the energy level used (Fig. 3b). Thus, X-ray beams with three different energy levels, 33.0, 33.2 and 33.4 keV were generated and tested for their efficacy to destruct tumor spheroids. The results of this analysis are shown in Fig. 4 which shows effect of monochromatic X-ray irradiation on tumor spheroids loaded with IPO. As can be seen, irradiation with monochromatic X-ray of 33.2 keV decreased the size of tumor spheroids significantly after irradiation for 30 min. In contrast, irradiation with 33.0 keV X-ray did not cause destruction of tumor spheroids. Interestingly, a monochromatic X-ray of 33.4 keV was much less effective in spheroid destruction compared with the X-ray of 33.2 keV; although the size of spheroids is decreased, significant portion of the spheroids remained after the exposure and incubation.

### Apoptosis induction during spheroid destruction

**Figure 5:**
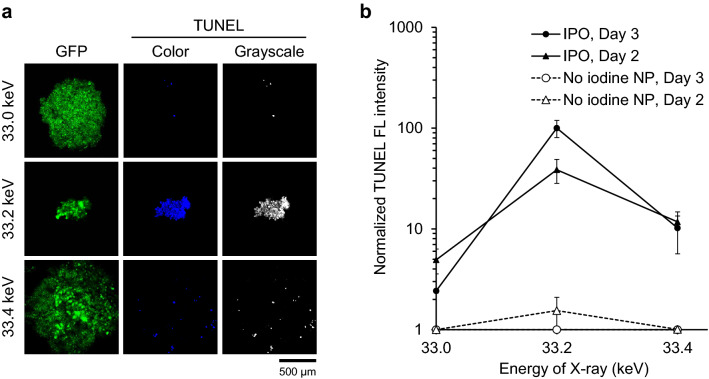
Detection of apoptosis in tumor spheroids by TUNEL assay. OVCAR8 tumor spheroids incubated with IPO were irradiated with 33.0, 33.2 and 33.4 keV monochromatic X-rays for 30 min. After irradiation, spheroids were incubated for 2 or 3 days in a CO_2_ incubator and cells were collected and TUNEL staining was performed. (**a**) TUNEL image of OVCAR8 tumor spheroids incubated for 3 days after IPO loading followed by the X-ray irradiation. Images of TUNEL reflecting apoptosis are displayed by blue (color) and white (grayscale). (**b**) Quantification of TUNEL signal. Data after 2-day or 3-day incubation are presented as mean ± s.e.m. (*n* = 3). *FL* fluorescence.

The destruction of tumor spheroids described above is likely due to apoptosis induction. In order to investigate this point, we carried out TUNEL (TdT-mediated dUTP nick end labeling) assay to detect DNA cleavage that occurs during apoptosis induction. Spheroids after irradiation with monochromatic X-rays followed by incubation in the CO_2_ incubator for 2 and 3 days were collected and processed for the TUNEL assay. Briefly, tumor spheroids were incubated with terminal deoxynucleotidyl transferase with Br-dUTP. Anti-BrdU antibody having Alexa fluorescence was used to examine incorporation of Br-dUTP. As shown in Fig. 5a, blue fluorescence was detected with tumor spheroids irradiated with 33.2 keV monochromatic X-ray. We also displayed the results in gray scale. TUNEL fluorescence per spheroid area was measured and plotted in Fig. 5b. As can be seen, we observe significant level of TUNEL signal after irradiation followed by 2 or 3-day incubation as summarized in Fig. 5b. Results of 2-day incubation are shown in Supplementary Fig. S7, and those of 3-day incubation are shown in Fig. 5a. The apoptosis occurs during the period of incubation after irradiation, as the manifestation of apoptosis requires extended period after the irradiation. Consistent with this idea, the TUNEL signal is increased after incubation for 3 days (Fig. 5b). The apoptosis induction was observed after irradiation with 33.2 keV X-ray, but much less with 33.0 keV X-ray. With 33.4 keV X-ray, apoptosis induction was also less. Almost no apoptosis was observed with the no iodine NP.

### DNA double strand breaks are induced by the irradiation

**Figure 6:**
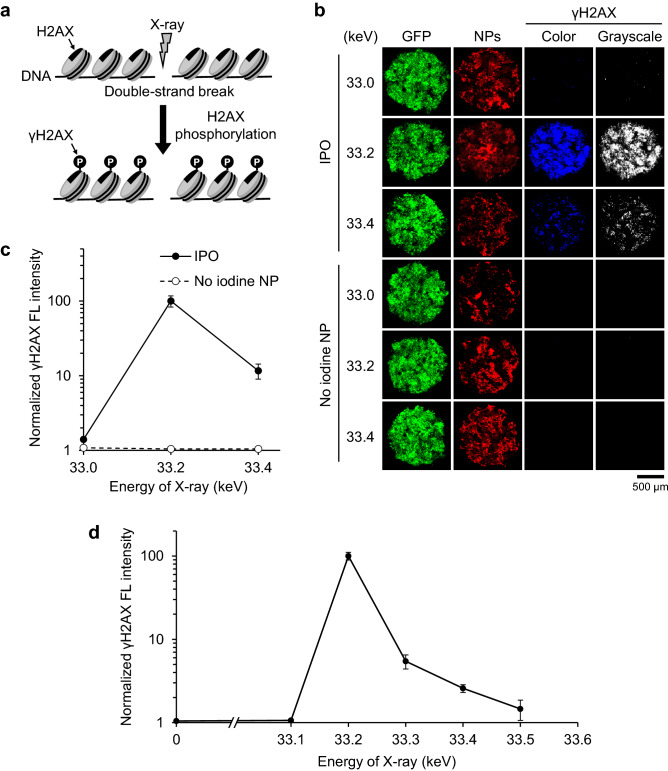
Detection of DNA double strand breaks in tumor spheroids. (**a**) An assay to detect double strand DNA breaks. (**b**) OVCAR8 tumor spheroids incubated with IPO or nanoparticles without iodine were irradiated with 33.0, 33.2 and 33.4 keV monochromatic X-rays for 30 min. After irradiation, spheroids were fixed immediately and γH2AX foci assay was performed. Images of γH2AX foci reflecting DNA double strand breaks are displayed by blue (color) as well as by white (grayscale). Tumor spheroids incubated with nanoparticles that do not contain iodine (no iodine NP) were used as no iodine controls. Red fluorescence shows nanoparticles (NPs) labeled with rhodamine-B. (**c**) Quantitation of double strand DNA breaks plotted against the energy of X-ray. Mean ± s.e.m. (*n* = 3). *FL* fluorescence. (**d**) Detection of double strand DNA breaks using X-rays with 0.1 keV interval. OVCAR8 tumor spheroids incubated with IPO were irradiated with monochromatic X-rays for 30 min. After irradiation, spheroids were fixed immediately and γH2AX foci assay was performed. Mean ± s.e.m. (*n* = 3). *FL* fluorescence.

Upon irradiation of iodine with monochromatic X-ray, it is expected that photoelectric effect will happen on iodine atoms and one of the consequences will be the release of the Auger electrons. These electrons have strong DNA damaging effect including double strand DNA cleavage. Therefore, we decided to investigate whether DNA double strand breaks are induced by the irradiation. This was examined by using γH2AX foci assay. This method detects DNA double strand breaks; upon the generation of double strand breaks, histone H2AX molecules are rapidly phosphorylated (Fig. 6a). Phosphorylation of Serine-139 is one of the earliest events upon X-ray exposure and this can be detected by the use of an antibody specific for this phosphorylation. We collected tumor spheroids right after the irradiation with monochromatic X-rays and processed for the γH2AX assay. As can be seen in Fig. 6b, positive signals as seen by blue fluorescence in our assay were detected with IPO loaded tumor spheroids after irradiation with 33.2 keV monochromatic X-ray. The signal was dependent on the presence of iodine, as little signal was detected with the no iodine NP. In contrast, the signal for DNA double strand breaks was not detected after irradiation with 33.0 keV X-ray. Note that the tumor spheroid distribution of control nanoparticle (no iodine NP) was very similar to that observed with IPO. Interestingly, double strand DNA break signal was much weaker after 33.4 keV irradiation compared with the signal observed after 33.2 keV irradiation (Fig. 6b,c). Note that the size of the spheroids is similar between all the samples, as they have not been incubated in CO_2_ incubator at this point and have not undergone apoptosis. These results provide convincing demonstration that double strand DNA breaks are induced in the IPO loaded spheroids and that the optimum level of DNA breaks was observed with 33.2 keV monochromatic X-ray. We further investigated the optimum effect of K-edge energy X-ray by varying energy at 0.1 keV interval and examining double strand DNA breaks. As can be seen in Fig. 6d and Supplementary Fig. S8, an optimum effect was observed with 33.2 keV X-ray. The effect was maximum with 33.2 keV X-ray but decreased as the energy increased to 33.3, 33.4 and 33.5 keV.

## Discussion

In this paper, we have demonstrated the ability of iodine-containing porous organosilica nanoparticles to cause destruction of tumor spheroids upon irradiation with monochromatic X-ray. The destruction was dependent on the presence of iodine, as little destruction was observed in the absence of iodine. Iodine was incorporated into the network of nanoparticles by the use of iodine-containing precursor for nanoparticle synthesis. The nanoparticle is also surface modified with phosphonate to enhance their high dispersibility throughout tumor spheroids. Indeed, confocal microscopy of the tumor spheroids after incubation with IPO demonstrated distribution of the nanoparticles throughout tumor spheroids. Our success in distributing IPO uniformly throughout the spheroids may be due to the phosphonate modification of our nanoparticles, as mesoporous silica-based nanoparticles with negative or positive charge were recently shown to penetrate into the core of tumor spheroids^38^. These nanomaterials have additional advantage to localize to perinuclear regions upon cellular uptake. Our IPO nanoparticles were also localized to the perinuclear regions in a cell (Supplementary Fig. S5). Penetration into tumor spheroids and the localization close to the nucleus where DNA resides are likely to contribute to dramatic effects of monochromatic X-ray irradiation. Finally, we note that IPO inhibits no major cytotoxicity as shown in Supplementary Fig. S6.

We have investigated the mechanism of tumor spheroid destruction. The spheroid destruction occurred during incubation of the spheroids for two to 3 days but not right after the irradiation. As shown in Fig. 6, the size of the spheroids did not change after the irradiation. The tumor destruction is due to apoptosis induction that takes place during incubation for 2–3 days in CO_2_ incubator, as revealed by the detection of the TUNEL signal. We have also shown that double-strand DNA breaks occur after the irradiation with monochromatic X-ray. This was possible by using the **γ**H2AX assay that detects H2AX phosphorylation. Therefore, it appears that the irradiation with monochromatic X-ray results in the generation of DNA double strand breaks that then induce apoptosis of cancer cells during incubation in CO_2_ incubator.

The effect of monochromatic X-ray irradiation on iodine occurred with the X-ray of 33.2 keV which corresponds to the energy just above the K-absorption edge energy of this element. Results of experiments using three different energy levels (33.0, 33.2 and 33.4 keV) demonstrated that spheroid destruction, apoptosis induction and double strand DNA breaks occurred with the 33.2 keV X-ray irradiation, but not with 33.0 keV irradiation and less with 33.4 keV irradiation. Further analysis using X-rays with 0.1 keV intervals confirmed the optimum effect of K-edge energy X-ray (33.2 keV). Thus, our observations point to the importance of the K-edge energy of iodine for the effect on tumor spheroids. These results are consistent with the photoelectric effects of iodine that are induced when X-ray of K-edge energy (33.2 keV) is used, as X-ray energy is absorbed by K-shell electrons. However, the energy absorption alone does not explain the decrease in efficiency of tumor spheroid destruction with X-rays having energy levels higher than the K-edge energy. Multiple events including the release of the Auger electrons happen after the K-edge energy absorption. The energy of electrons released may differ depending on the energy absorbed. It will be interesting to follow the Auger emission after irradiating with different energy X-rays. However, these experiments require extensive preparation, as the emission takes place inside the cell. Furthermore, the obtained results need to be analyzed by considering the energy dependencies of emitted Auger electrons that are obtained by theoretical calculations. Thus, we plan to investigate the mechanism behind the IPO action after sufficient preparations are completed. We are also planning to test the ability of IPO to inhibit tumor growth in mouse models in the future.

The ability of iodine to induce cell killing upon X-ray irradiation has been investigated in a variety of studies^12, 39–41^. In earlier studies, iodine was introduced into cancer cell DNA by the use of iododeoxyuridine and the cells were irradiated with monochromatic X-ray^12, 39–41^. The enhancement of cell killing by the irradiation of monochromatic X-ray was relatively low (up to two-fold)^12^. The dramatic results we observed may be due to the use of tumor spheroids, as the earlier studies used suspended cancer cells that are sensitive to X-ray irradiation even without iodine. Hainfeld et al^22^ developed very small nanoparticles (20  nm) consisting of a crosslinked triiodobenzene molecule with a PEG coating. These nanoparticles have prolonged blood half-life and exhibited enhancement of radiation therapy in animal model systems^22^. A recent report shows that these nanoparticles bind collagen I mediated by PEG^42^ and thus they appear to differ from the nanoparticles we developed in terms of its material and the mode of action. Various types of iodine-containing nanoparticles are expected to be developed in the future.

## Methods

### Chemicals

Rhodamine-B isothiocyanate (RITC, mixed isomers), Cetyltrimethylammonium bromide (CTAB, 98%), 3-(trihydroxysilyl)propyl methylphosphonate monosodium salt solution (50% in H_2_O) and (3-iodopropyl)trimethoxysilane (IPTMS, ≥ 95%) were provided by Sigma-Aldrich. 1,2-bis(triethoxysilyl)ethane (BTSE, > 95%) and bis[3-(triethoxysilyl)propyl]tetrasulfide (BTESPTS) was obtained from Fluorochem; 8 M NaOH aqueous solution, ammonium nitrate (≥ 98%) and 3-aminopropyltriethoxysilane (APTES, 99%) from Nacalai Tesque company. Water with Milli Q grade and ethanol 99.5% from Wako were used.

### Synthesis and characterization of iodine-containing porous organosilica (IPO)

Preparation of IPO was carried out by following synthesis of nanoparticles as previously reported^32^ with modifications. A mixture of CTAB (250 mg, 685.9 μmol) and NaOH aqueous solution (125 μL of 8 M solution, 4 mmol) in water (120 mL) was vigorously stirred (1400 rpm) at 80 °C. RITC (2.5 mg, 4.7 μmol) was dissolved in 5 mL ethanol followed by addition of APTES (6 μL, 25.6 μmol). The mixture was agitated for 30 min at room temperature. BTSE (300 μL, 809.7 μmol) was then added to the solution and stirred for an additional 5 min. Then, the silane solution was added dropwise to the CTAB solution prior to immediate BTESPTS (100 μL, 203.2 μmol) addition. After 15 min of reaction, IPTMS (500 μL, 2.5 mmol) was added dropwise followed by 3-(trihydroxysilyl)propyl methylphosphonate monosodium aqueous solution (315 μL, 827.9 μmol) 15 min after the IPTMS. The reaction was then held at 80 °C under 1400 rpm agitation for 2 h. The solid material was collected by centrifugation and washed three times with ethanol. To remove CTAB, the as-synthesized solids were overnight refluxed in a solution of 0.3 g of ammonium nitrate and ethanol (50 mL) with 1200 rpm agitation at 80 °C. The IPO products were centrifuged, washed three times with ethanol and dried overnight at ambient pressure and temperature. IPO: pink powder. IR (ν, cm^−1^): 1032 (Si–O–Si), 2890 (CH_2,s_), 2925 (CH_2,as._). Zeta potential (IPO, 25 °C, pH = 7.5): − 42.9 mV. Nanoparticles that do not contain iodine (no iodine NP) were synthesized in the same way as that used to synthesize IPO except that the iodine precursor was not included. IR (ν, cm^−1^): 1032 (Si–O–Si), 2894 (CH_2,s_), 2925 (CH_2,as._). Zeta potential (no iodine NP, 25 °C, pH = 7.5): − 37.5 mV.

Scanning Electron Microscopy (SEM) images were performed on a JEOL JSM-75FCT. Transmission Electron Microscopy (TEM) images were performed on a JEOL JEM-2100F. The scanning transmission electron microscopy-energy dispersive X-ray (STEM-EDX) analysis was carried out with a JEOL JEM-2200FS + JED2300T system operated at 200 kV. DLS was performed using a Zetasizer μV Malvern apparatus (ZMV2000). Zeta-potential measurements were carried out on a Malvern Zetasizer Nano-ZS at controlled temperature (25 °C) and at different pH conditions (2, 4, and 7.5). Low-pressure N_2_ adsorption measurements at 77 K were carried out on a Quantachrome Autosorb iQ volumetric gas adsorption analyzer. Helium was used as estimation of dead space. Ultrahigh-purity-grade N_2_, and He (99.999% purity) were used throughout adsorption experiments. ICPE-9000 Shimadzu was used for the ICP-AES analysis that quantitated the amount of iodine in the nanoparticle.

### Tumor spheroids and incubation with IPO

Tumor spheroids were formed by using human ovarian cancer cells OVCAR8 (ATCC) expressing green fluorescent protein (GFP) grown in RPMI1640 medium supplemented with 10% heat-inactivated FBS and 1% penicillin/streptomycin. 5.0 × 10^3^ cells were placed in a well of PrimeSurface 96U culture plate (MS-9096U, Sumitomo Bakelite Co., LTD., Japan). Cells were cultured at 37 °C in a humidified CO_2_ for about 7 days. The diameter of the tumor spheroid was around 500 µm. Tumor spheroids were incubated with rhodamine-B labeled IPO (2 µg IPO which contains 66 ng of iodine was incubated with a tumor spheroid in 200 µL of culture media) for 24 h at 37 °C in a humidified CO_2_ incubator. After collecting each spheroid in a PCR tube, the tube was centrifuged at 1500 rpm for 5 min. The spheroids were washed with ice-cold PBS and fixed overnight with 4% paraformaldehyde at 4 °C. For Hoechst staining, the spheroids were treated with 99.8% methanol for 30 min at − 80 °C. After washing, they were stained with Hoechst 33258 solution for 30 min in dark for confocal microscopy.

### Cell uptake of IPO

OVCAR8 cells were seeded at 5.0 × 10^3^ in a 35 mm glass-based dish (Iwaki) in 190 µL medium and incubated at 37 °C in a humidified CO_2_. Twenty-four hours later, 10 µL of IPO dispersed in water was added to the medium. After incubation for 24 h, the cells were washed with ice-cold PBS and fixed overnight with 4% paraformaldehyde at 4 °C. Before Hoechst staining, the cells were treated with 99.8% methanol for 30 min at − 80 °C. After washing, the cells were stained with Hoechst 33258 solution for 30 min in dark for confocal microscopy.

### Cytotoxicity assay of IPO

OVCAR8 cells were seeded in 96-well flat bottom plates at 5.0 × 10^3^ or 1.0 × 10^3^ cells per well in 190 µL of medium and incubated at 37 °C in a humidified CO_2_. Twenty-four hours later, 10 µL of IPO dispersed in water was added to the medium. After incubation for 1 or 3 days, WST-8 assay was performed using Cell Counting Kit-8 (Dojindo) according to the instruction manual.

### Synchrotron X-ray generation and irradiation

Synchrotron radiation X-rays were generated from a bending magnet source. Synchrotron radiation white X-rays were monochromatized by a double-crystal fixed-exit monochromator with silicon 311 crystals. The SPring-8 storage ring was operated in top-up mode with a constant storage ring current of 100 mA. Monochromatized X-rays were shaped by a horizontal and a vertical slit. The incident beam size was 0.7 mm in height and 1.4 mm in width at the sample position. Two ion chambers were placed on the optical axis to monitor the incident beam intensity. Energy calibration of synchrotron radiation X-rays was done by measuring X-ray K-edge absorption profile of potassium iodide film, which was placed between the ion chambers. X-ray absorption *μt* was calculated by dividing *I* by *I*_0_, where *μ*, *t*, *I*, and *I*_0_ were linear absorption coefficient of iodide, thickness of iodide, the transmitted X-ray intensity, and the incident X-ray intensity, respectively.

Tubes containing spheroids were placed in a sample rack specially designed for the present experiment. The rack was located on XYZ stage, which enables us to move samples on the optical axis for irradiation without entering the experimental hatch; series of irradiations can be done automatically. The sample position was checked before irradiation by using optical microscope and laser. It was also monitored by an X-ray CCD camera during irradiations. It is worth mentioning here that the high energies of X-rays did not enable the absorption contrast of the spheroids nor tubes to be observed by the CCD camera. Refraction-enhanced X-ray images of the tubes were obtained to monitor the sample positions.

### TUNEL assay

Spheroids were washed with ice-cold PBS and overnight fixed with 4% paraformaldehyde at 4 °C. After washing twice with PBS, the spheroids were permeabilized with PBS containing 0.2% Triton X-100 for 30 min at room temperature. TUNEL staining was performed using APO-BRDU Apoptosis Kit (NBP2-31161, Novus). After washing the spheroids twice with Wash Buffer, they were incubated in DNA Labeling Solution containing bromolated deoxyuridine triphosphate nucleotides (Br-dUTP) and terminal deoxynucleotidyl transferase (TdT) for 2 h at 37 °C with agitation every 15 min. After washing 5 times with Rinse Buffer, the spheroids were incubated in Antibody Solution containing Alexa 350-labeled rabbit anti-BrdU polyclonal antibody (bs-0489R-A350, Bioss) at dilution of 1:100 overnight at 4 °C in the dark. After washing the spheroids with PBS containing 0.1% Triton X-100 and 5 mg/mL BSA, they were stained their nuclei with DRAQ5 solution [PBS, 0.1% Triton X-100, 5 mg/mL BSA and 10 µM DRAQ5 (BioStatus)] for 30 min in the dark. The spheroids were washed 3 times with PBS containing 0.1% Triton X-100 and 5 mg/mL BSA and then observed under a Nikon A1 laser scanning confocal microscope (Nikon). TUNEL fluorescence intensity and spheroid area were quantified using ImageJ software (NIH). The spheroid areas were determined from bright-field images. TUNEL fluorescence intensity per spheroid area was detected and quantified.

### DNA double strand break detection

Irradiated spheroids were immediately fixed with 4% paraformaldehyde overnight at 4 °C. After washing twice with PBS, the spheroids were permeabilized with PBS containing 0.2% Triton X-100 for 30 min at room temperature. After washing the spheroids twice with PBS containing 0.1% Triton X-100 and 5 mg/mL BSA, they were incubated in primary antibody solution [PBS, 0.1% Triton X-100, 5 mg/mL BSA and anti-γH2AX rabbit mAb (#9718, Cell Signaling Technology, 1:1000)] overnight at 4 °C. After washing 5 times, the spheroids were incubated in secondary antibody solution [PBS, 0.1% Triton X-100, 5 mg/mL BSA and Alexa 350-labeled goat anti-rabbit IgG (#A-11046, Thermo Fisher Scientific, 1:1000)] overnight at 4 °C in the dark. After washing the spheroids with PBS containing 0.1% Triton X-100 and 5 mg/mL BSA, they were stained their nuclei with DRAQ5 solution [PBS, 10 µM DRAQ5 (BioStatus), 0.1% Triton X-100, 5 mg/mL BSA and] for 30 min in the dark. The spheroids were washed 3 times with PBS containing 0.1% Triton X-100 and 5 mg/mL BSA and then observed under a Nikon A1 laser scanning confocal microscope (Nikon). γH2AX fluorescence intensity per spheroid area was detected and quantified.

## Supplementary Information

Supplementary Information.

## Data Availability

Materials and data obtained in this study are deposited to the publisher site and are available upon request.
