# Supplementary material for: Iodine containing porous organosilica nanoparticles trigger tumor spheroids destruction upon monochromatic X-ray irradiation: DNA breaks and K-edge energy X-ray
Source: Sci Rep. 2021 Jul 14;11:14192. doi: 10.1038/s41598-021-93429-9 (PMC8280225; doi:10.1038/s41598-021-93429-9)
Supplement: Supplementary file 1 — Supplementary Information. [file 41598_2021_93429_MOESM1_ESM.pdf]

Supplementary Information

Iodine containing porous organosilica nanoparticles trigger tumor spheroids destruction upon monochromatic X-ray irradiation: DNA breaks and K-edge energy X-ray

Yuya Higashi[1], Kotaro Matsumoto[1], Hiroyuki Saitoh[2], Ayumi Shiro[2], Yue Ma[1], Mathilde Laird[1], Shanmugavel Chinnathambi[1], Albane Birault[1], Tan Le Hoang Doan[3], Ryo Yasuda[2], Toshiki Tajima[4], Tetsuya Kawachi[2] and Fuyuhiko Tamanoi[1,*]

[1]Institute for Integrated Cell-Material Sciences, Institute for Advanced Study, Kyoto University, Kyoto, Japan

[2]Kansai Photon Science Institute, Quantum Beam Science Research Directorate, National Institutes for Quantum and Radiological Science and Technology, Hyogo, Japan

[3]Center for Innovative Materials and Architectures, Vietnam National University-Ho Chi Minh City, Ho Chi Minh City, Vietnam

[4]Department of Physics and Astronomy, University of California, Irvine, CA, USA

[*]Correspondence: tamanoi.fuyuhiko.2c@kyoto-u.ac.jp

1. Comparison of IPO and no iodine nanoparticles (no iodine NP)

IPO nanoparticles and the no iodine NP (which were synthesized without iodine precursors and used as a control) were characterized. Their size, chemical composition, surface charge as well as their tumor spheroid distribution were studied. The comparison is summarized in Supplementary Table S1 and presented in corresponding Figures. Based on these results, we conclude that no significant difference in NP property and spheroid uptake can be attributed to the addition of the IPTMS precursor.

Supplementary Table S1. Comparison of IPO and no iodine NP

Analysis	IPO	No iodine NP	Indicated Figure
Average size by SEM	80 nm	80 nm	Fig. S1a
Average size by TEM	80 nm	80 nm	Fig. S1b
Zeta potential	−42.9 mV	−37.5 mV	Fig. S2
FT-IR	Si-O-Si bond<br>-CH_2- bond	Si-O-Si bond<br>-CH_2- bond	Fig. 1d
Tumor spheroid distribution	Uniform throughout	Uniform throughout	Fig. 6b

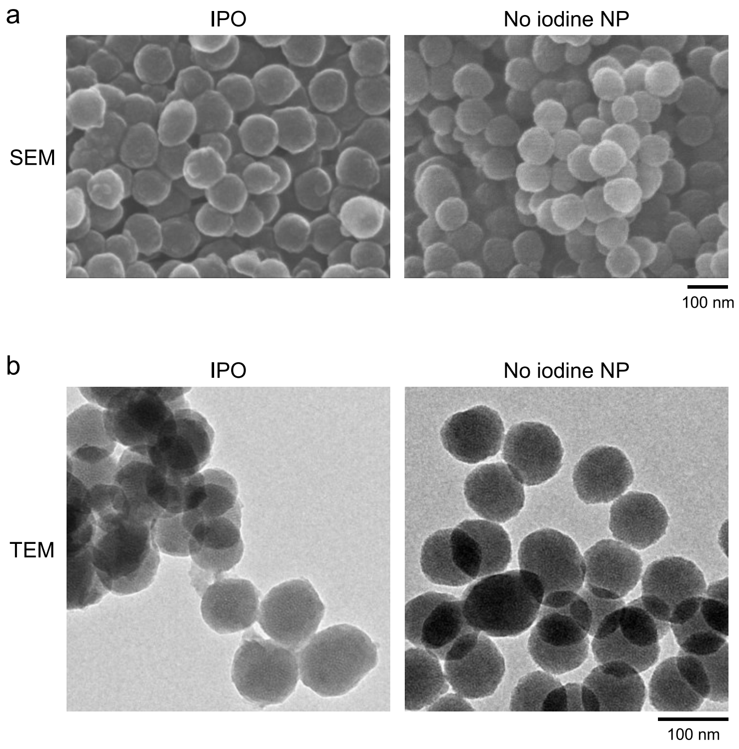

Supplementary Figure S1. SEM (**a**) and TEM (**b**) images of IPO nanoparticles and no iodine NP.

2. Zeta potential measurements

We have examined zeta potential of IPO (+I, phosphonated), no iodine NP (−I, phosphonated) as well as that of bare NP (−I, non-phosphonated) at a controlled temperature of 25 °C and at three different pH values (2, 4 and 7.5). As shown in Supplementary Fig. S2, at pH 7.5, the NPs have similar surface charge with a zeta potential value around −40 mV. Nevertheless, in acidic pH different behaviors are observed. Indeed, at pH 2, phosphonated silica NP (IPO and no iodine control) still exhibit negative zeta potential while bare silica NP shows a positive zeta potential. This clearly evidences that the isoelectric point (IEP) of the phosphonated NP (IEP < 2) have changed compared to the bare silica nanoparticle (IEP > 2) due to successful phophonation of the NP surface.

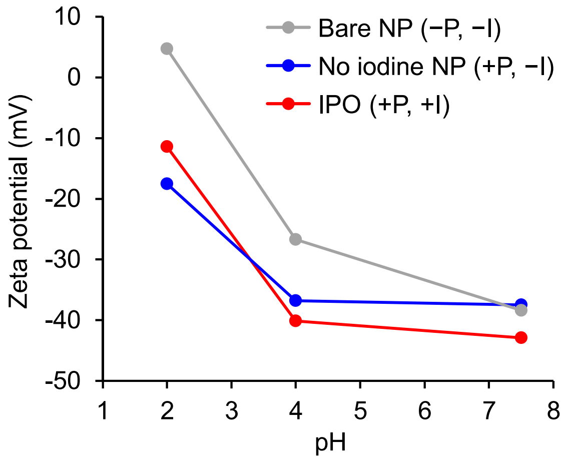

Supplementary Figure S2. Zeta potential of IPO, no iodine NP and bare NP at different pH (25 °C).

3. Further characterization of IPO

3.1. Nitrogen adsorption-desorption of IPO

IPO nanoparticles were further characterized by the nitrogen adsorption-desorption isotherm analysis shown in Supplementary Fig. S3. The results reveal the presence of a small quantity of micro and mesopores, also reflected by a specific surface area of 67 $m^2.g^{-1}$. The absorption occurring over a large range of P/P_0 suggests that a large variety of pore size is present. The large increase of absorbed gas at high P/P_0 may be attributed to interparticular space between the nanoparticles. The presence of unorganized pores is consistent with the results of TEM analysis (Fig. 1c).

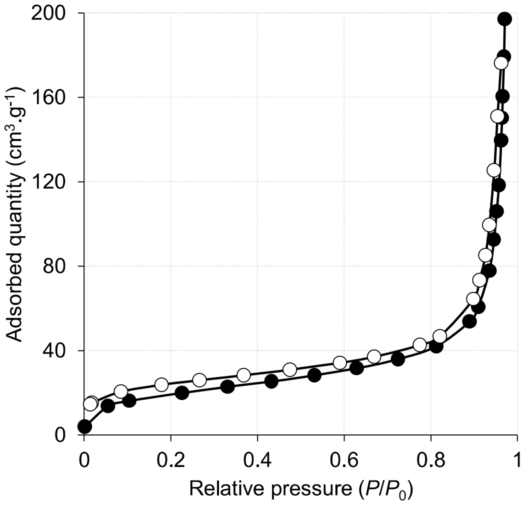

Supplementary Figure S3. Nitrogen adsorption-desorption analysis of IPO nanoparticles. The black circle and white circle represent adsorption and desorption, respectively.

3.2. Colloidal stability of IPO

Colloidal stability of IPO was examined by carrying out DLS measurement. As shown in Supplementary Fig. S4, a sharp peak around 80 nm was detected. When the measurement was carried out in the presence of BSA, the peak shifted slightly to larger particle size. We believe that this is due to the formation of protein corona on the nanoparticle.

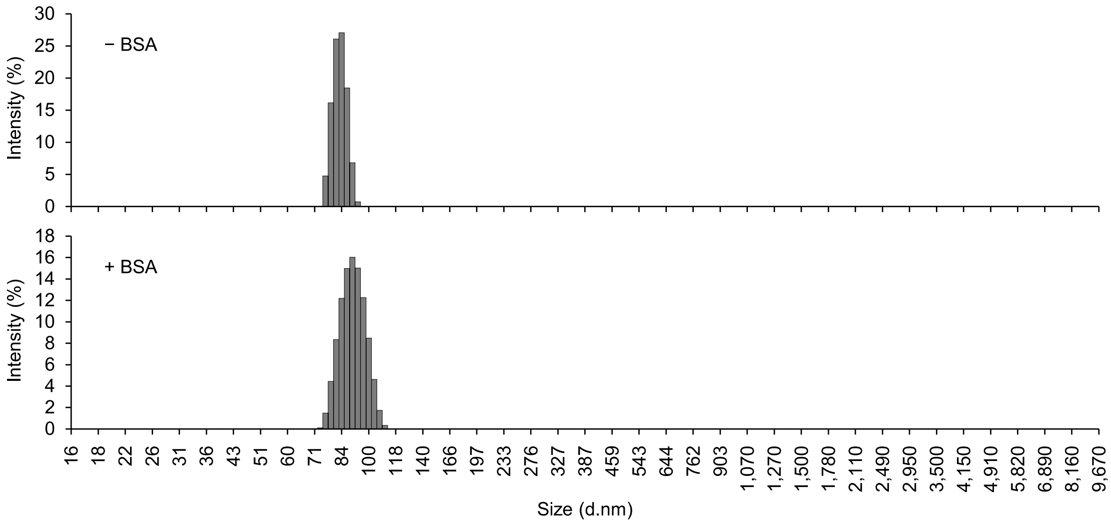

Supplementary Figure S4. DLS analysis of IPO dispersed in water or PBS at pH 7.4 with 0.1% BSA.

3.3. Uptake of IPO into human cancer cells

IPO nanoparticles (rhodamine-B labeled) at a concentration of 25 μg/mL were incubated with OVCAR8 cells for 24 h, whose cells are engineered to express GFP (Supplementary Fig. S5). The cells were washed and examined by confocal microscopy. As can be seen, red fluorescence of the nanoparticles was detected at the periphery of cell nucleus. The lower figure shows localization of IPO nanoparticles at a single plane. In this case, fluorescence of GFP, nuclear stain and nanoparticles are merged so that the nanoparticles are seen as yellow fluorescence.

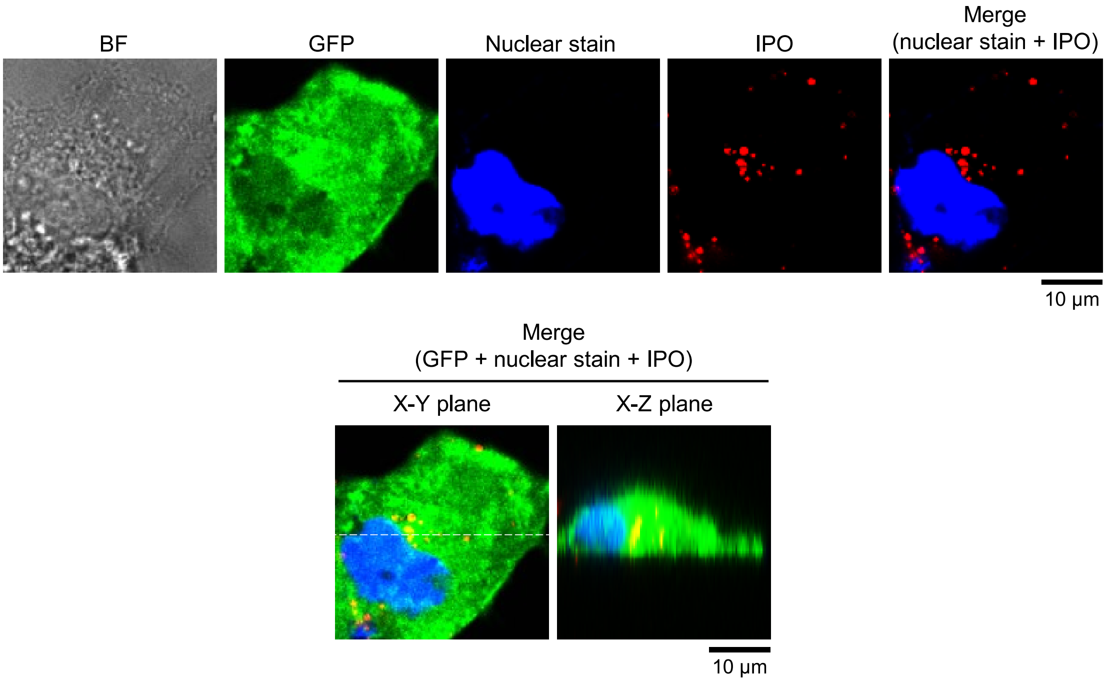

Supplementary Figure S5. Uptake of IPO into OVCAR8 cell. The fluorescence of IPO labeled with rhodamine-B is shown in red. Nucleus was stained with Hoechst dye. Green fluorescence shows GFP of OVCAR8 cell. The white dashed line indicates the X-Z plane. BF, bright field.

3.4. Cytotoxicity of IPO

IPO nanoparticles do not exhibit cytotoxic effect on cells. This was examined by incubating OVCAR8 cells with varying concentrations of IPO. No major cytotoxicity was detected as shown in Supplementary Fig. S6.

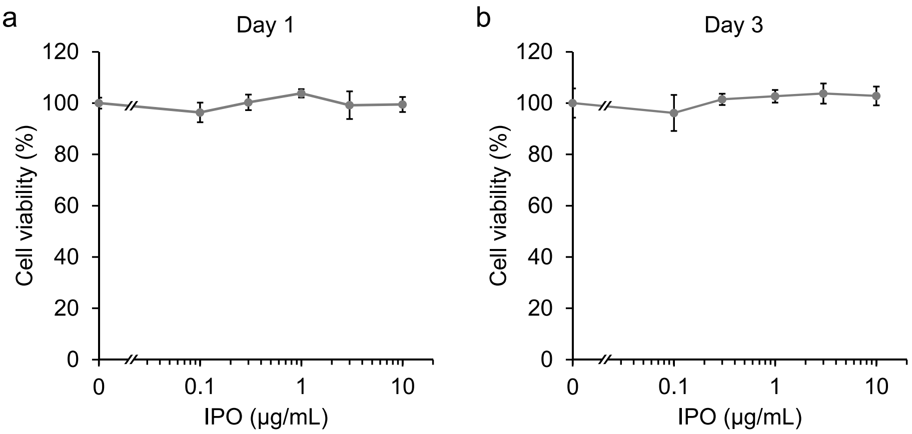

Supplementary Figure S6. WST-8 assay of OVCAR8 cells at Day 1 (**a**) and Day 3 (**b**) after IPO loading. Mean ± s.d. (n = 4).

4. Monochromatic X-ray irradiation of IPO-loaded tumor spheroids

4.1. Detection of apoptosis

To detect apoptosis in nanoparticle-loaded tumor spheroids after monochromatic X-ray irradiation, the TUNEL assay was performed. We observed the high level of TUNEL signal in IPO-loaded tumor spheroid after irradiation with 33.2 keV followed by 2-day incubation (Supplementary Fig. S7).

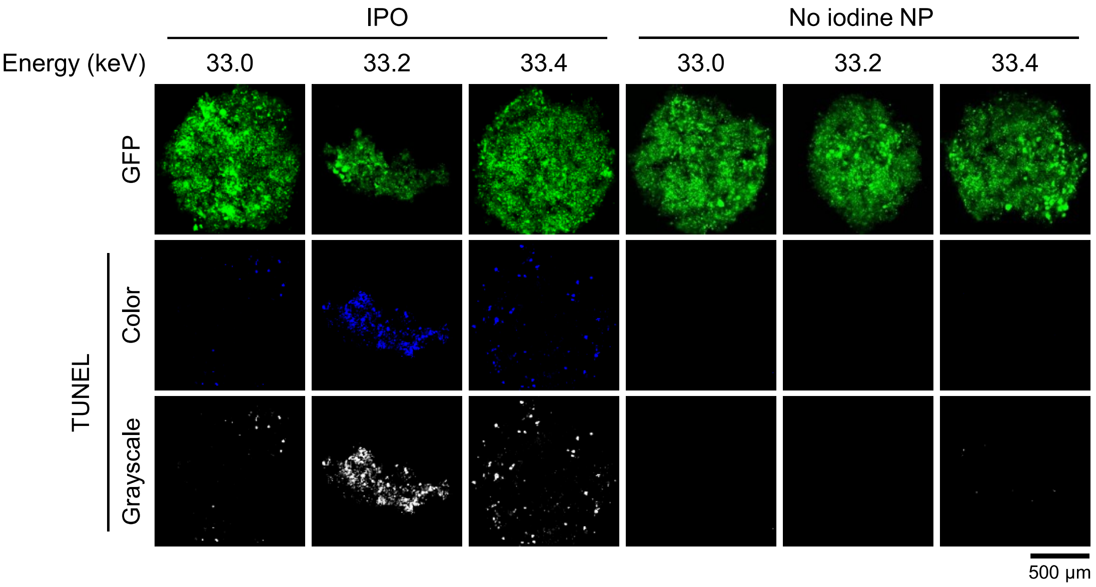

Supplementary Figure S7. Detection of apoptosis in tumor spheroids by TUNEL assay 2 days after X-ray irradiation. OVCAR8 tumor spheroids incubated with IPO or control nanoparticles without iodine (no iodine NP) were irradiated with monochromatic X-rays of indicated energies for 30 min. After irradiation, spheroids were incubated for 2 days in a CO_2 incubator and TUNEL staining was performed. Images of TUNEL reflecting apoptosis are displayed by blue (color) as well as by white (grayscale).

4.2. Detection of DNA double strand breaks

In order to investigate the optimum effect of K-edge energy X-ray by varying energy at 0.1 keV interval, we investigated DNA double strand breaks in IPO-loaded tumor spheroids after irradiation. As can be seen in Supplementary Fig. S8, an optimum effect was observed with a 33.2 keV X-ray.

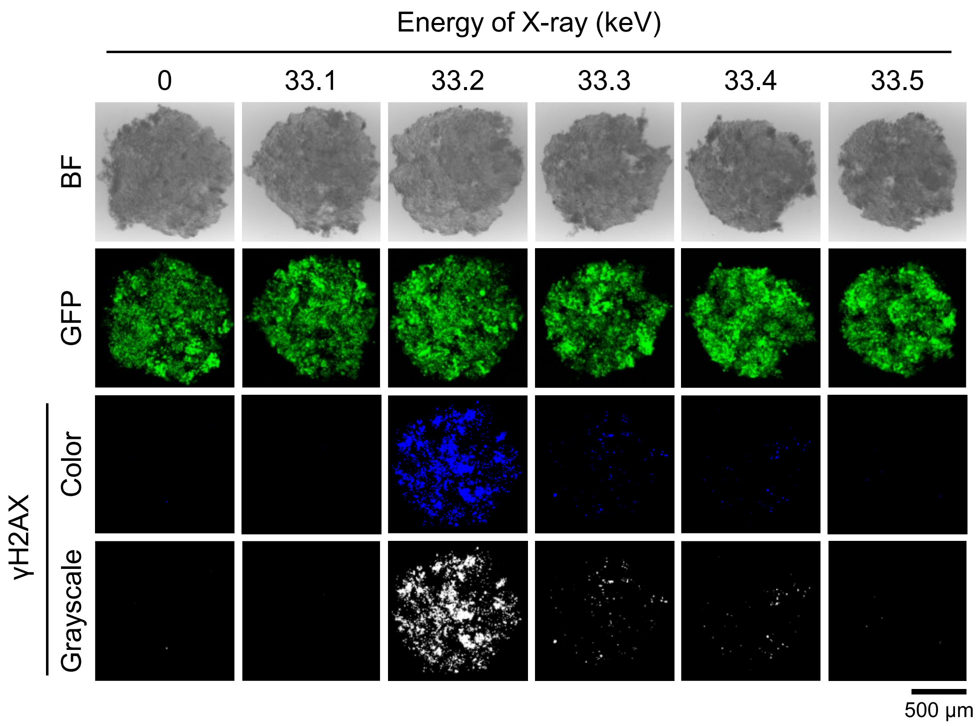

Supplementary Figure S8. Detection of double strand DNA breaks in tumor spheroids using X-rays with 0.1 keV interval. OVCAR8 tumor spheroids incubated with IPO were irradiated with monochromatic X-rays of indicated energies for 30 min. After irradiation, spheroids were fixed immediately and γH2AX foci assay was performed. Images of γH2AX foci reflecting DNA double strand breaks are displayed by blue (color) as well as by white (grayscale). BF, bright field.
